# Anti–Melanoma Differentiation Associated Gene 5 (MDA 5) Dermatomyositis Complicated by Spontaneous Pneumomediastinum: A Case Report From South Africa

**DOI:** 10.1002/ccr3.9573

**Published:** 2024-11-18

**Authors:** M. Myburgh

**Affiliations:** ^1^ Department of Internal Medicine, Kalafong Provincial Tertiary Hospital Pretoria University of Pretoria Pretoria South Africa

**Keywords:** dermatomyositis, MDA 5 DM, rheumatology, spontaneous pneumomediastinum

## Abstract

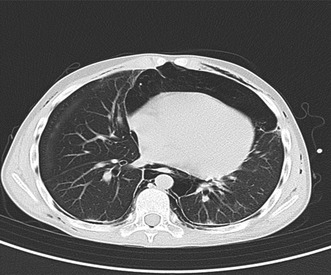

**Summary**

- Spontaneous pneumomediastinum is a rare complication in anti–melanoma differentiation associated gene 5 (MDA 5) dermatomyositis (DM).
- In this case, it occurred relatively early in the course of this patient's illness before established interstitial lung disease was present; hence, it should be investigated in any anti‐MDA 5 DM patient with dyspnea or chest pain.

## 1 Introduction

Anti–melanoma differentiation associated gene 5 (MDA 5) dermatomyositis (DM) is a recently described subtype of DM, which is usually clinically amyopathic and presents with unique mucocutaneous signs and may complicate with rapidly progressive interstitial lung disease (RP‐ILD) in as many as 69% of cases [1].

We describe a case of a 14‐year‐old male from Pretoria, South Africa, who presented with progressive ulcerating cutaneous lesions, who later on complicated with a rare pulmonary manifestation associated with anti‐MDA 5 DM.

Anti‐MDA 5 DM is an uncommon subtype of idiopathic inflammatory myositis. It presents with unique cutaneous manifestations and is commonly complicated by a potentially life‐threatening RP‐ILD [2].

Diagnosis of this subtype of DM is challenging and may result in delayed therapy. The presentation is characteristically clinically amyopathic with no proximal muscle weakness or elevated muscle enzymes, together with striking ulcerating cutaneous lesions not typically found in classic DM [3].

The condition is considered to be driven by an occlusive vasculopathy as well as a cytokine response to the autoantigen MDA 5, which is involved in innate immune response against various viruses including a potential link to COVID‐19 as a possible trigger [4, 5, 6]. MDA‐5 is a cytoplasmic retinoic acid inducible gene I (RIG I)‐like receptor that functions as part of the innate immune system [2]. It recognizes viral nucleic acids and triggers type 1 interferon production halting viral replication. Epidemiological data from Japan revealed a cluster of anti‐MDA 5 DM cases in rural areas around the Kiso River with a link to Coxsackie virus in the water from October to March. Similar clustering was detected around the Yangtze river in China [4].

These patients present with cutaneous ulcerations as the hallmark of the disease, driven by a vasculopathy. The common areas involved include the MCP and IP joints, elbows, knees, and nail folds [3].

Palmar papules, often termed inverse Gottron's papules, may also be a unique feature occurring often on the creases. Lateral digit hyperkeratosis or scaling is also encountered along with alopecia and oral ulcers [3].

The systemic features typically encountered during the course of the disease include arthritis and fever. The majority of patients are clinically amyopathic. ILD is also a hallmark of the illness, with the risk of life‐threatening rapidly progressive ILD occurring [2, 3]. Spontaneous pneumomediastinum has also been described [7].

## 2 Case History/Examination

**FIGURE 1:**
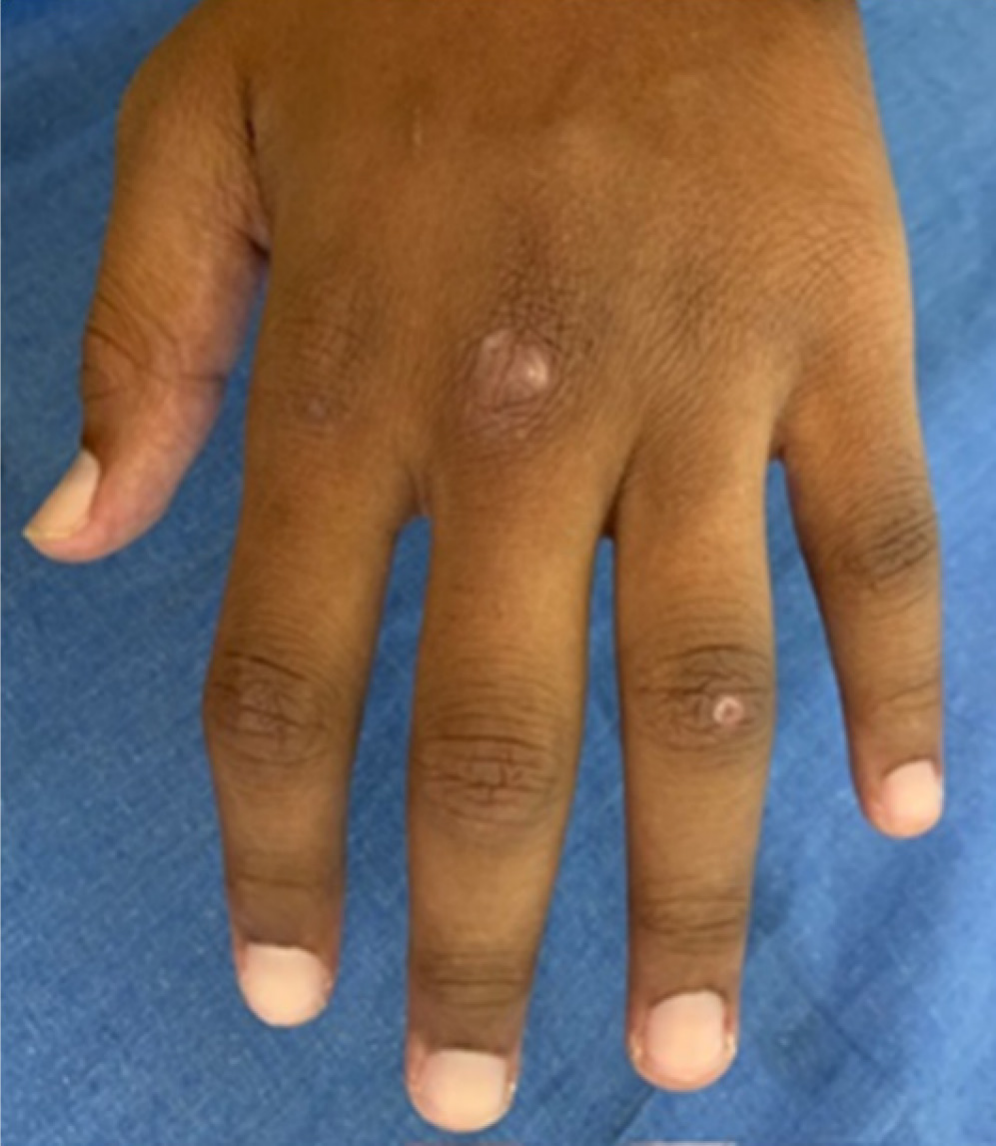
Lesions at index presentation.

A month before the COVID 19 pandemic in South Africa a 14‐year old male presented to our Rheumatology clinic complaining of progressive arthritis with swelling of the small joints of the hands and both knees. This was associated with worsening fatigue and difficulty playing football. Upon examination, not only synovitis of the affected joints was evident but also small ulcerating lesions were noted bilaterally and symmetrically over the third MCP joints (Figure 1). Fundoscope‐capillaroscopy was normal. Clinically, there was no evidence of myopathy. The cardiovascular and respiratory examination were normal.

## 3 Investigations/Treatment

Initial blood work‐up revealed a negative ANA, ENA, anti‐Jo, anti‐Mi, and anti–PM‐Scl. RF and anti‐CCP were negative as well as antiphospholipid antibodies. Complement was normal and creatinine kinase was not elevated. Full blood count revealed a leucopenia of 2.49 × 10^9^ /L. Renal function and liver functions were unremarkable. CRP was < 10 mg/L with an ESR of 17 mm/h. At this point, the myositis immunoblot assay was not available to us at our National Health Laboratory.

The ulcerating skin lesions raised a concern for vasculitis, so the patient was initiated on azathioprine at 2 mg/kg/day and a short course of prednisone at 1 mg/kg/day. The myositis immunoblot assay was, however, eventually arranged through a private laboratory, which revealed a high positive titer for anti‐MDA 5 antibodies. The immunoblot assay also revealed mid‐positive anti–Ro 52 antibody and a low‐positive anti–PM‐Scl 75 antibody; however, the patient showed no clinical features suggesting systemic sclerosis [8].

Response to therapy at 2 months was unsatisfactory and cyclosporin at 2 mg/kg/day was added to his regimen. The ulcers continued to enlarge and the joint deformities progressed. A decision was made to start Rituximab 500 mg IVI. Shortly after this, the patient complicated with a septic arthritis of his knee. He underwent surgical drainage for this on more than one occasion and during this septic phase of his illness received immunomodulation with IVIG every 4–6 weeks.

Once the sepsis had resolved, the patient was continued on a regimen of mycophenolate mofetil 1 g BD with cyclosporin 2 mg/kg/day, low‐dose prednisone as well as rituximab 6 monthly. The initial dose of rituximab was 500 mg administered twice in a 2‐week period, and subsequent doses at follow‐up were 1 g maintenance doses. The patient received a total of 5 doses of rituximab and now is on a maintenance of mycophenolate mofetil 500 mg BD and cyclosporin 75 mg BD po.

**FIGURE 2:**
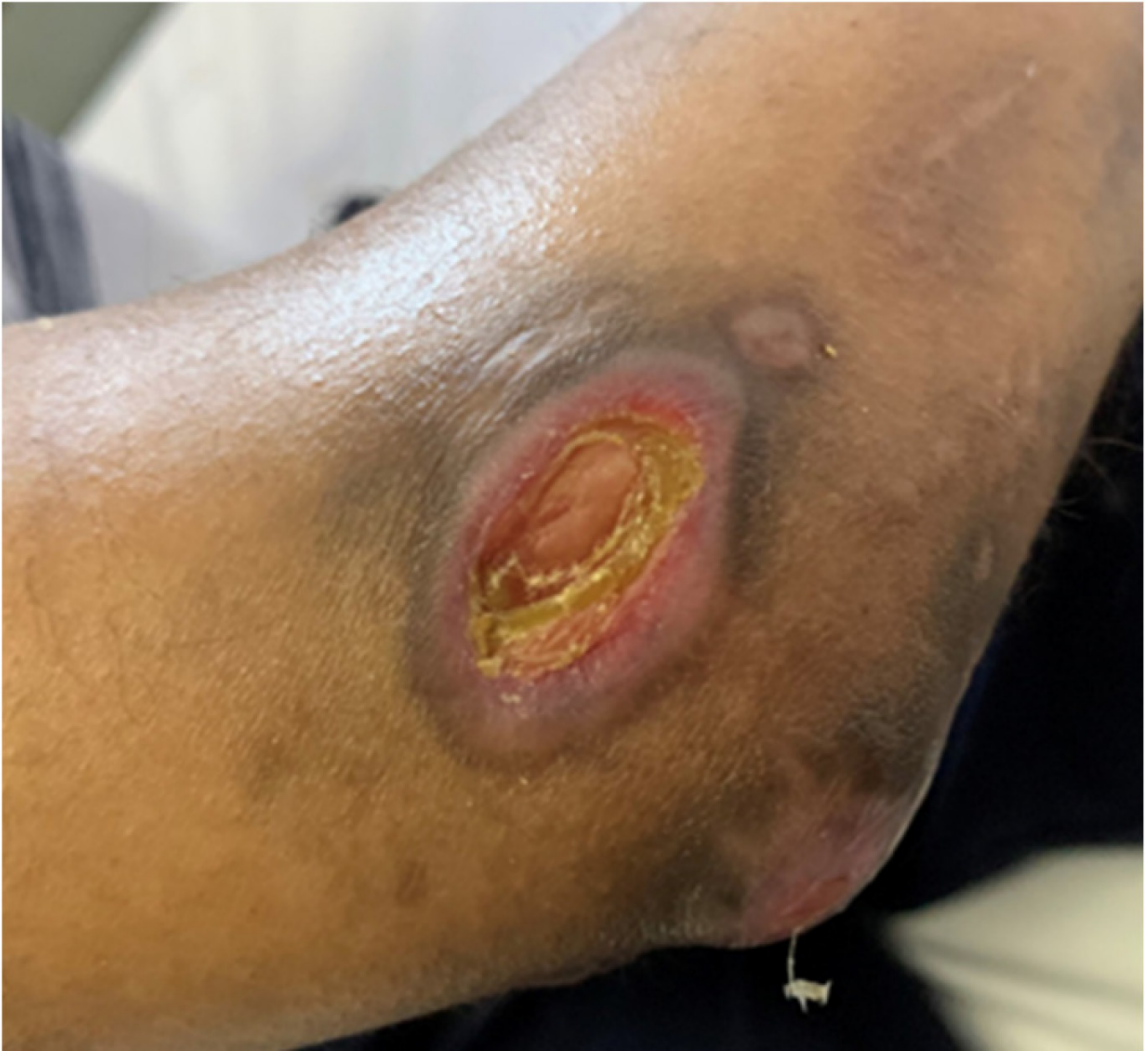
Healing ulcer elbow.

**FIGURE 3:**
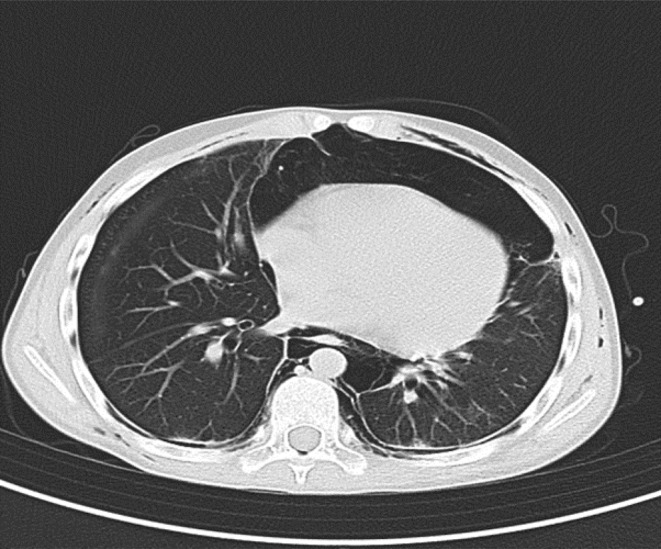
Spontaneous pneumomediastinum.

**FIGURE 4:**
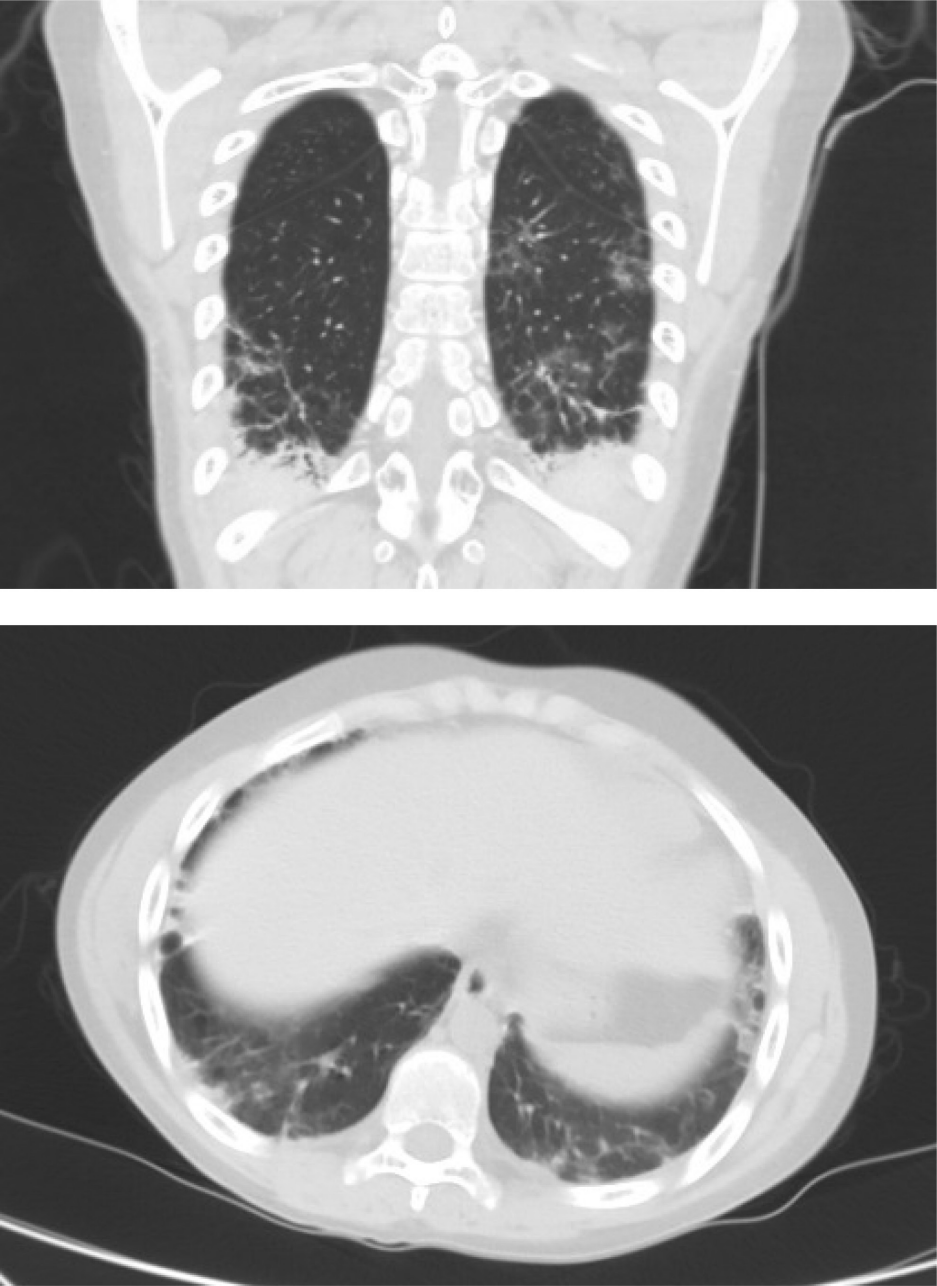
Probable UIP.

During this period, the cutaneous lesions slowly improved as depicted below (Figure 2), at which point he suddenly developed acute chest pain and dyspnea. The patient had developed a spontaneous pneumomediastinum (Figure 3). This is a well‐documented complication of anti‐MDA 5 DM and is thought to arise from a vasculopathy of the bronchial vessels. The pneumomediastinum did not complicate and resolved with conservative therapy after a few weeks. In addition to the massive pneumomediastinum, the CT scan revealed early signs of ILD with bilateral posterior basal lower lobe, subpleural, interlobular septal thickening, and intralobular lines with bilateral posterior basal pleural thickening. Eight months later on repeat imaging there was mild but definite evidence of ILD with bilateral peripheral, subpleural predominant fibrotic changes present with mild traction bronchiectasis in keeping with a probable or indeterminate usual interstitial pneumonitis (Figure 4).

Timeline of events.

**Table:** 

Month 1	Month 6	Month 8	Month 9	Month 16	Month 24
Digital ulcers and arthritis	Septic arthritis	Spontaneous pneumomediastinum	Resolution of pneumomediastinum and gradual improvement in skin ulcers	Probable UIP on imaging	Stability and gradual improvement with healed skin lesions

## 4 Conclusion

**FIGURE 5:**
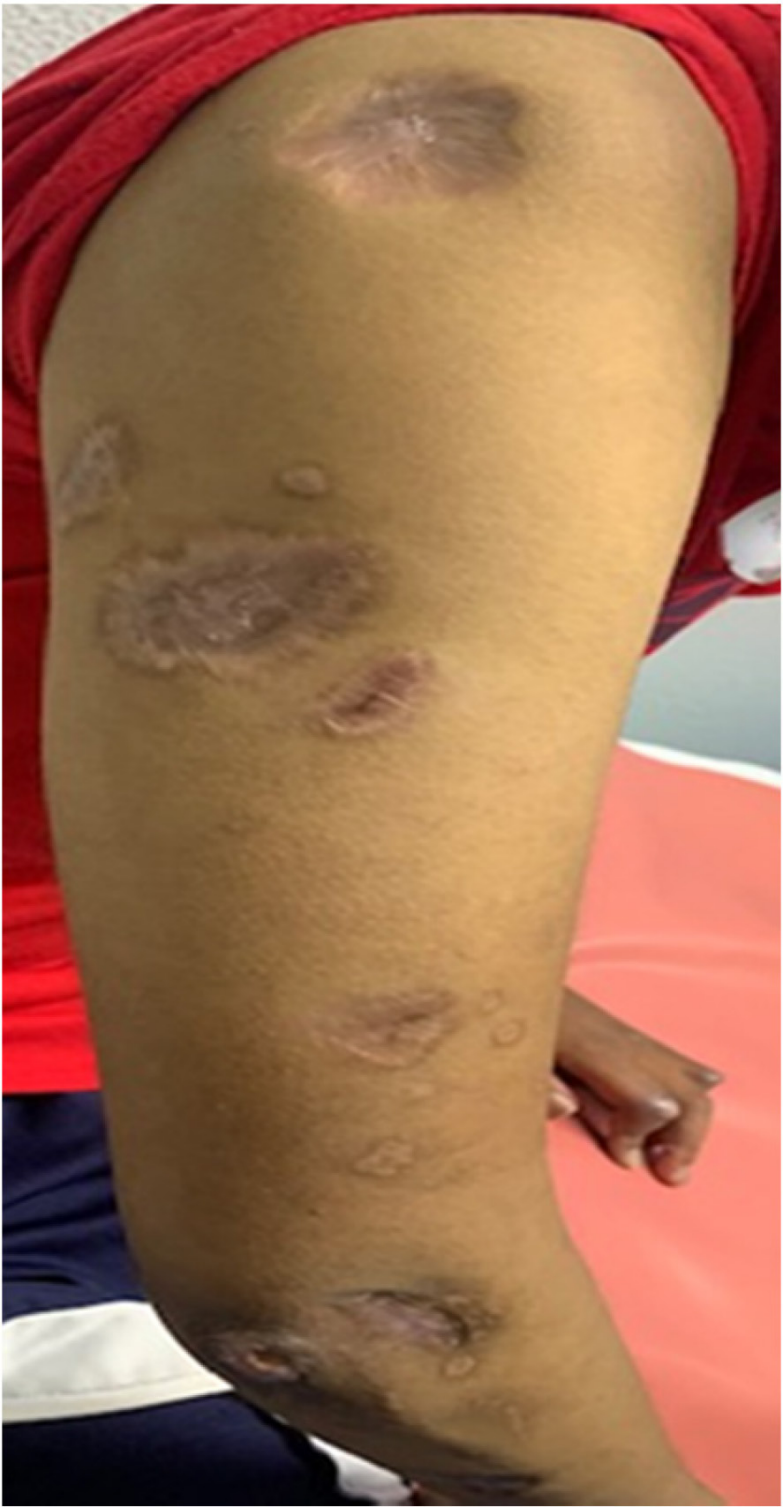
Healed ulcers.

The patient has continued to improve on the current regimen with gradual tapering of prednisone. The ulcers have healed as depicted in Figure 5.

This was the first anti‐MDA 5 DM patient, confirmed on the immunoblot assay, managed at our Rheumatology clinic. I am pleased to say we have now acquired myositis immunoblot assays at our National Health Laboratory Services, which is aiding us in diagnosing and phenotyping these IIM patients more effectively and hopefully resulting in better directed therapy.

## 5 Discussion

Anti‐MDA 5 DM is a novel and fascinating condition, which is currently still not well defined. Spontaneous pneumomediastinum in MDA 5 DM is well described and is often associated with a worse prognosis and more often with rapidly progressive ILD. The incidence of spontaneous pneumomediastinum in MDA 5 DM is around 15% with a mortality rate as high as 60% [9, 10]. Spontaneous pneumomediastinum may occur due to air leakage through non‐partitional alveolar walls and subsequent medial dissection along pulmonary vessels to the mediastinum; however, the mechanism in DM is not well understood. In this patient, the pneumomediastinum occurred at a time when minimal ILD was present. Months later despite some progression of his ILD he remained well and showed no signs of developing a rapidly progressive ILD. As the mechanism of this complication is poorly understood, we can only speculate as to which form of therapy would be best to prevent and treat this life‐threatening complication. Increased awareness of MDA 5 DM and its complications are crucial in improving outcomes in this potentially devastating illness.

## Author Contributions

**M. Myburgh:** writing – original draft.

## Consent

Written informed consent was obtained from the patient to publish this report in accordance with the journal's patient consent policy.

## Data Availability

Data that support the findings of this case report are available from the corresponding author upon reasonable request.
